# Optimization of Pt(II) and Pt(IV) Adsorption from a Water Solution on Biochar Originating from Honeycomb Biomass

**DOI:** 10.3390/molecules29020547

**Published:** 2024-01-22

**Authors:** Kinga Morlo, Rafał Olchowski, Ryszard Dobrowolski

**Affiliations:** 1 Department of Analytical Chemistry, Institute of Chemical Sciences, Faculty of Chemistry, Maria Curie-Sklodowska University, M. C. Sklodowska Sq. 3, 20-031 Lublin, Poland; kinga.morlo1@gmail.com; 2 Department of Pharmacology, Toxicology and Environmental Protection, Faculty of Veterinary Medicine, University of Life Sciences, Akademicka Sq. 12, 20-950 Lublin, Poland; rafal.olchowski@up.lublin.pl

**Keywords:** platinum, automobile catalysts, slumgum-based biocarbon, leachate, adsorption mechanism

## Abstract

Novel CO_2_- and H_3_PO_4_-modified biochars were successfully synthesized from raw honeycomb biomass. They were characterized via several instrumental techniques. The optimal Pt(II) and Pt(IV) adsorption onto the studied biochars was reached for the initial pH of 1.5 and a contact time of 5 min (Pt(II)) and 24–48 h (Pt(IV)). The highest static adsorption capacities for Pt(II) and Pt(IV) were obtained for the H_3_PO_4_-modified biochar: 47 mg g^−1^ and 35 mg g^−1^, respectively. The Freundlich model described the Pt(II) adsorption isotherms onto both materials and the Pt(IV) adsorption isotherm onto the CO_2_-activated material, and the Langmuir model was the best fitted to the Pt(IV) adsorption isotherm onto the H_3_PO_4_-activated biochar. The best medium for the quantitative desorption of the Pt form from the H_3_PO_4_-modified biochar was 1 mol L^−1^ thiourea in 1 mol L^−1^ HCl. The adsorption mechanism of both the studied ions onto the synthesized H_3_PO_4_-modified biochar was complex and should be further investigated. The H_3_PO_4_-modified biochar was successfully applied for the first time for Pt(IV) removal from a spent automotive catalyst leaching solution.

## 1. Introduction

Platinum group metals (PGMs) are precious elements with some unique properties: splendid color, good electrical conductivity, high chemical resistance, high ductility and strong catalytic activity. Thus, they are widely applied in various areas of the economy, such as jewelry manufacturing, the chemical industry, electronic devices and automotive catalytic converters. PGMs have a high economic importance, so their global demand is still rising (above 230 t/year for Pt). Unfortunately, the natural source deposits of PGMs are very limited (66,000 t), and they are located in regions with high geopolitical risks (South Africa, Russia, Zimbabwe). The restricted reserve distributions of PGMs influence the limited supply of PGMs from primary resources (above 180 t/year for Pt). These factors cause the great significance of PGMs’ efficient recovery from secondary resources [1,2,3].

The main secondary source of Pt is spent automotive catalytic converters (SACCs) (43% in 2022). They contain about 1000 times higher amounts of Pt than natural ores. In Europe, the SACC industry is growing larger due to the implementation of the Euro 5 and Euro 6 emission standards and other regulations. Pt sourcing from SACCs can reduce waste disposal, limit power consumption, and lower environmental pollution. The skeleton of an SACC has a honeycomb structure made of cordierite (mixed magnesium, aluminum and silicon oxides) coated with a thin layer of the washcoat (90% γ-Al_2_O_3_), PGMs and other additives. The precious metal (Pt/Pd/Rh or Pt/Rh) load in SACCs is between 0.1% and 0.5% (about 3–7 g of Pt in one catalytic converter). The Pt and Pd present in the SACCs catalyze the oxidation reactions of CO and hydrocarbons to CO_2_ and H_2_O. Additionally, Rh is the catalyzer of NO reduction to N_2_. The recovery of Pt from SACCs is a complex process, which consists of the initial pretreatment (mechanical processing, calcination, chemical processing), an enrichment step (hydrometallurgy, pyrometallurgy or biometallurgy) and a separation/refining step (precipitation, electrolysis, extraction or adsorption) [4].

Usually, pretreatment processes are applied with respect to increasing the leaching efficiency of PGMs by obtaining the highest dissolution rate, kinetics and minimalizing the cost of the process. Pretreatment also helps to avoid the use of aggressive solvents. The main chemical operations involved in pretreatment are the following: reduction roasting, oxidative roasting, alloying and sustainable processes from the environmental and economical points of view. It is worth emphasizing that the hydrometallurgical proceeding represents an innovative and promising method for PGM recycling; however, its use in the industry is rather infrequent [1,2,4].

Recently, a hydrometallurgical process was proposed to recover platinum from spent diesel catalysts, based on a first leaching step with solutions under mild conditions using H_2_O_2_ in an acidic medium using HCl, followed by the adsorption of platinum species on granular activated carbon. The leaching process is carried out by the oxidation of metal platinum from the surface of the catalyst by active oxygen derived from the decomposition of the oxidant (H_2_O_2_) in an acidic medium. Chlorides act as ligands to stabilize the Pt(II) ions in solution. Since chloride can come from both the acidifying medium and the appropriate chloride salt, the source of chloride can be, e.g., NaCl, NaOCl, or CuCl, as presented by Yakoumis et al. [1]. These approaches seem to be remarkable because they lead to the complete recovery of platinum with negligible extraction of other metals from the catalyst. From the environmental point of view, this process is advantageous due to reducing the safety risks associated with the use of concentrated HCl and the emissions of NO_x_ deriving from the use of nitrogen species in conventional leaching. From an ecological point of view, this process leads to a reduction in the risks associated with the use of concentrated HCl and NO_x_ emissions when using aqua regia in the leaching process. Summarizing, as a result of hydrometallurgical processes, Pt(IV) ions are leached, usually in the form of chloride complexes [1,4].

It is worth emphasizing that the rapid development of metallurgy, electroplating, and the chemical industry has resulted in the worldwide consumption of hundreds of thousands of tons of Pt(CN)_4_^2−^ cyanide annually. As a result of these activities, large amount of wastewater containing metal–cyanide complexes must be treated. This wastewater must be purified before it is released into the environment, with the simultaneous recovery of platinum. Such an approach reduces the harm to the environment and forms the circular economy of precious metals. Various treatment methods, including photocatalysis, ion exchange, oxidation methods, biodegradation and active carbon adsorption, have been extensively explored for removing platinum–cyanide complexes from industrial wastewater. The limitations of the commonly used porous solid adsorbents for the removal of platinum–cyanide complexes include the exhibited low adsorption capacity, poor selectivity, and difficulties with regeneration [5]. In summary, adsorption is the most popular process used for Pt recovery both from SACC leachates and platinum–cyanide complexes in industrial wastewater. It is known as an economical and eco-friendly method.

Furthermore, it provides efficient metal ion extraction from solutions containing its low concentrations. Hazardous organic solvents can be avoided by using the solid adsorbents. Additionally, the adsorption of Pt onto the surface of solid matrices is an interesting opportunity to produce new catalysts through the appropriate treatment. Various porous solids can be used for this purpose, such as chelating resins, polymers, metal–organic frameworks, biopolymers, supported ionic liquids, self-assembled monolayers (SAMs) of 1-(11-mercaptoundecyl)imidazole (Im-C11-SH), 12-mercaptododecanoic acid (MDDA) on Au-coated Si and glass chips, chitosan or carbon materials. The latter ones are especially interesting due to their unique properties: large surface area, which can be easily chemically changed for efficient and selective Pt removal; thermal conductivity; chemical stability; excellent electrical conductivity and availability. Among carbon materials, the biochars are more and more popular because they possess the advantages of carbonaceous materials and their production can be determined as upcycling the biowaste feedstocks [6,7,8,9,10].

Honeycomb biomass is an interesting source of biochar because it contains about 50% wt. of carbon. Thousands of tons of this biowaste are produced globally each year. It contains both biohazard components (such as bacteria) and toxic organic/inorganic substances (e.g., Cr, Ni, Amitraz). Inappropriate storage of such waste can lead to serious environmental pollution. Up to now, there has been no recycling of the honeycomb biomass. The production of the biochar from honeycomb biomass can partially solve this problem. Moreover, the organics present in this biomass can be the source of heteroatoms (O, N), being the selective active species toward Pt on the surface of the made biochar [11,12,13].

In the literature, both CO_2_ and H_3_PO_4_ were used for biochar modification. CO_2_-physical modification is based on the Boudouard reaction, which is thermodynamically favorable at temperatures above 710 °C. This activation method mainly promotes the pore formation and improves the microporous structure, which results in the high specific surface area and pore volume of the final material. Kim et al. [14] synthesized oak-wood-derived biochar under N_2_ and CO_2_ atmospheres, and the obtained products resulted in the specific surface area of 231 m^2^ g^−1^ and 464 m^2^ g^−1^ for the N_2_ and CO_2_ atmospheres, respectively. The efficiency of the CO_2_ modification of biochar is related to the different types of biomass used for its production. For example, CO_2_-activated biochars derived from cellulose, lignin and grass had a specific surface area of 515 m^2^ g^−1^, 402 m^2^ g^−1^ and 25 m^2^ g^−1^, respectively. Additionally, during the CO_2_ activation process, the enhanced release of volatile organic species was observed. CO_2_ is an efficient, non-corrosive, oxidizing and pore-forming agent, which provides an inexpensive and operationally easy activation process. On the contrary, the H_3_PO_4_ modification of biochar is a composed and temperature-dependent process, and it influence both the surface chemistry and porosity of the final material. This acid acts as the catalyzer, which promotes the bond cleavage and formation of cross-links via cyclization and condensation reactions. During the modification process, the phosphates, polyphosphate bridges bonded to the oxygen species and volatile compounds (e.g., CO_2_, H_2_O) are formed. The highest P content (5.8–12.0 wt. %) in the carbonaceous material can be obtained at 800 °C. Furthermore, H_3_PO_4_-modified biochar heated above 450 °C can reduce its porosity via the process of dilation, which is influenced by the phosphate groups. Researchers [15] produced biochar from pine sawdust under an N_2_ atmosphere and next modified it with H_3_PO_4_. The specific surface area increased from 408 m^2^ g^−1^ to 900 m^2^ g^−1^, and O and P were introduced to the material surface with simultaneous N removal due to the modification process. The H_3_PO_4_-modification requires the washing-off step of the final material and influences its catalytic and redox properties [16,17,18,19,20]. 

In our present work, the honeycomb-made biochar was for the first time applied for the efficient Pt recovery from the leachate of SACCs. Raw honeycomb material was thermally modified using CO_2_ or H_3_PO_4_. The obtained biochar materials were characterized via physicochemical techniques. Additionally, Pt(II) and Pt(IV) optimization of adsorption experiments on the obtained biochars from aqueous solutions were conducted. The studies included the pH influence on their adsorption ability, contact time and desorption with respect to the differentiated leaching medium. Finally, the adsorption isotherms were prepared and fitted to theoretical models. The most effective biochar for platinum species removal after leaching from ERM^®^-EB504a material was proposed. Based on the experimental data, a mechanism for the adsorption of platinum ions on selected biocarbon was chosen.

## 2. Results and Discussion

### 2.1. Physicochemical Characteristics

Both obtained biochars possess a porous structure with a high specific surface area (especially BC_H_3_PO_4_ with 608 m^2^ g^−1^) and a total pore volume above 0.1 cm^3^ g^−1^. The BC_CO_2_ and BC_H_3_PO_4_ materials have also narrow mesopores (3.7–3.9 nm) (Table 1). The high specific surface area values and the presence of mesopores indicate the micro–mesoporous structure of both synthesized slumgum-originated biochars. According to these data, the H_3_PO_4_ was a more efficient porous agent than the CO_2_. In the presence of H_3_PO_4_ and at a high temperature, some chemical reactions can take place within the carbon precursor, which leads to the formation of volatile products. These gaseous products can penetrate the structure of the carbon precursor and can be the reason for pore creation in the biochar material [16].

**Figure 1:**
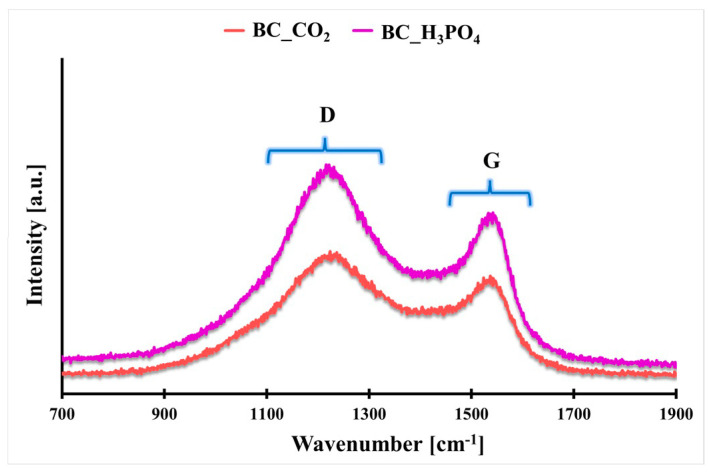
The Raman spectra of the synthesized biochars with the marked D and G bands.

In Figure 1, the Raman spectra recorded for the BC_CO_2_ and BC_H_3_PO_4_ materials are presented. In both cases, two specific bands are present: D and G bands. The D band located at 1328 cm^−1^ is related to the aromatic carbon rings confined in the defective graphene domains. The G band located at 1595 cm^−1^ is characteristic of in-plane carbon sp^2^ bonds stretching. The I_D_/I_G_ ratio indicates whether the sample structure is closer to graphite or amorphic carbon. For the BC_CO_2_ and BC_H_3_PO_4_ biochars, the I_D_/I_G_ ratios are 1.25 and 1.32, respectively. The I_D_/I_G_ values above 1.0 indicate the amorphic structure of the carbonaceous material, like in the activated carbon, with defects possibly originating from heteroatoms such as O, N or P [21]. Thus, it can be concluded that both synthesized biochars possess an amorphic structure with randomly organized graphene domains. More defects are observed in the case of the BC_H_3_PO_4_ biochar, which confirms the incorporation of more heteroatoms into the graphene domains in this case, contrary to the BC_CO_2_ biochar.

In Table 1, the zeta potential values of both studied biochars are also presented. In each case, there is a negative zeta potential, which can be related to the higher population of negatively charged functionalities than positively charged on the biochar surface. The negatively charged functionalities can originate from mainly carboxylic and/or phosphoric acid groups, which can be easily dissociated in an aqueous environment. A higher absolute value of the zeta potential was observed for the BC_H_3_PO_4_ (−28.6 mV) than for the BC_CO_2_ (−4.22 mV). The BC_H_3_PO_4_ surface can have more carboxylic and phosphoric acid functionalities than the BC_CO_2_ surface. Thus, the BC_H_3_PO_4_ can efficiently adsorb positively charged species, such as metal cations.

**Figure 2:**
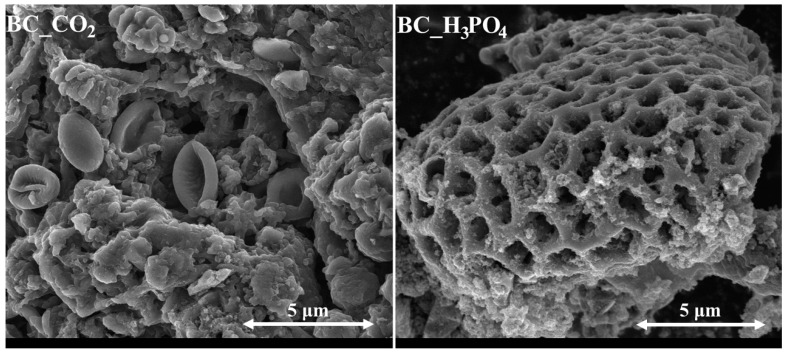
SEM microphotographs of the studied biochars (magn. 10,000×).

In Figure 2, the SEM microphotographs of the studied biochars are presented. For both biochars, their particles can be characterized by the complex heterogeneous morphology, which originates from the biological forms present in the pristine slumgum material, such as bee residues or honeybee silk [22,23]. In each case, pores with various sizes and shapes can be observed, which highlights the different nature of the porous structure of the studied biochars. 

The elemental composition of the studied biochars is presented in Table 2. In both cases, the synthesized materials possess a carbon content above 50 wt. %, which is evidence of its carbonaceous nature. In the structure of the BC_CO_2_ and BC_H_3_PO_4_ materials, there are also heteroatoms, such as oxygen, nitrogen, phosphorous, silicon, chlorine and sulfur, and metals, like potassium, calcium, iron and magnesium. These elements can originate from waxes, pesticides, microorganisms, larvae residues, dead bees and beehive frames present in the original slumgum material. More oxygen and phosphorous content is observed for the biochar activated by H_3_PO_4_ (O: 26.7–27.2 wt. %, P: 10.3–13.6 wt. %) than by CO_2_ (O: 9.06–23.8 wt. %, P: 0.29–1.4 wt. %). This can be related to the partial incorporation of orthophosphoric acid groups into the structure of the BC_H_3_PO_4_ biochar during its synthesis. Additionally, the elemental composition is more heterogeneous for the CO_2_-activated biochar than for the H_3_PO_4_-modified biochar. The higher content of O (23.8 wt. %), P (1.4 wt. %) and K (2.3 wt. %) is observed on the surface of the BC_CO_2_ biochar (XPS data) rather than in the deeper layers of the material (O: 9.06 wt. %, P: 0.29 wt. %, K: 0.43 wt. %) (SEM-EDS data). A different situation is observed for C and N, which are mainly observed inside the BC_CO_2_ biochar particles (C: 83.0 wt. %, N: 3.97 wt. %) rather than on their surface (C: 70.0 wt. %, N: 2.5 wt. %). Si is present in both biochars (BC_CO_2_: 2.87 wt. %, BC_H_3_PO_4_: 1.85 wt. %). S and Cl are not observed for the H_3_PO_4_-modified biochar, which can indicate that these elements were removed from the material during the H_3_PO_4_ high-temperature treatment. There are also traces of Mg and Ca present in both studied biochars. Fe is observed in the BC_CO_2_ (6.91 wt. %) and BC_H_3_PO_4_ (1.25 wt. %) biochars, which originates from the honeybee frames. The lower Fe content in the case of the BC_H_3_PO_4_ biochar can be related to its probable removal from the material during the distilled water washing of the orthophosphoric acid excess. Elements present on the studied biochar surface can constitute the binding sites for heavy metals (e.g., Pt). 

For the studied biochars, the high resolution XPS spectra were recorded. This included the C 1s, O 1s, N 1s and P 2p core energy levels. The obtained XPS spectra were deconvoluted, which revealed some signals for each core energy level: C 1s band (C=C sp^2^ at 284.3 eV, C-C/C-H sp^3^ at 285.5 eV, C-O at 286.7 eV, C=O at 287.8 eV and O=C-O at 289.9 eV), O 1s band (O=C/O=C-O/O-P at 530.9 eV, C-OH/O=P at 532.4 eV, O=C-O/P-OH at 533.7 eV and adsorbed O_2_/H_2_O at 535.5 eV), N 1s band (NH_2_ at 398.8 eV, quaternary nitrogen at 401.1 eV and N-O at 402.8 eV) and P 2p band (PO_4_^3−^ at 133.7 eV (P 2p_3/2_ part of P 2p doublet signal)) [20,24,25,26].

The highest content of C=C sp^2^ groups was observed for the BC_H_3_PO_4_ biochar (56.3 wt. %). This suggests that H_3_PO_4_ modification of the pristine slumgum material results in a more efficient carbonization process, with more carbon aromatic domains produced than in the case of the BC_CO_2_ biochar. The aliphatic carbon content is similar in both studied materials (29.4–30.3 wt. %). The same situation was observed for the carbonyl groups (1.2–1.8 wt. %). However, the C-O and O=C-O content was higher for the BC_CO_2_ biochar than for the BC_H_3_PO_4_ (9.3 wt. % and 7.0 wt. %; 8.3 wt. % and 5.2 wt. %, respectively). These clear changes can be the result of the orthophosphoric acid residues incorporation into the biochar surface modified in that manner. The same trend was observed for the signals obtained via O 1s deconvolution (O=C/O=C-O/P-O: 24.7 wt. % (BC_CO_2_) and 5.8 wt. % (BC_H_3_PO_4_); C-OH/P=O: 57.3 wt. % (BC_CO_2_) and 39.3 wt. % (BC_H_3_PO_4_); O=C-O/P-OH: 17.4 wt. % (BC_CO_2_) and 54.2 wt. % (BC_H_3_PO_4_); adsorbed O_2_/H_2_O: 0.6 wt. % (BC_CO_2_) and 0.7 wt. % (BC_H_3_PO_4_)). The presence of acidic functional groups originating from H_3_PO_4_ (PO_4_^3−^) on the BC_H_3_PO_4_ surface was also confirmed by the P 2p deconvoluted signal. Differences in the nitrogen surface group content depending on the slumgum modification procedure were also noticed (NH_2_: 39.9 wt. % (BC_CO_2_) and 18.4 wt. % (BC_H_3_PO_4_); the quaternary nitrogen: 41.1 wt. % (BC_CO_2_) and 64.6 wt. % (BC_H_3_PO_4_); N-O: 19.0 wt. % (BC_CO_2_) and 17.0 wt. % (BC_H_3_PO_4_)). The N-O group content for both studied materials was similar. On the BC_CO_2_ surface, the content of NH_2_ and quaternary nitrogen was similar (ca. 40 wt. %), but for the BC_H_3_PO_4_ surface, a higher content of quaternary nitrogen than NH_2_ groups was observed. This can be the result of better N incorporation into the graphene domains during H_3_PO_4_ modification rather than during CO_2_ activation. The built-in N in the graphene plains can act as a donor of electrons, which should promote the metal ions reduction by the carbonaceous surface [27]. Thus, the BC_H_3_PO_4_ biochar should possess better reduction properties than the BC_CO_2_ biochar.

### 2.2. Pt(II) and Pt(IV) Adsorption Optimization

**Figure 3:**
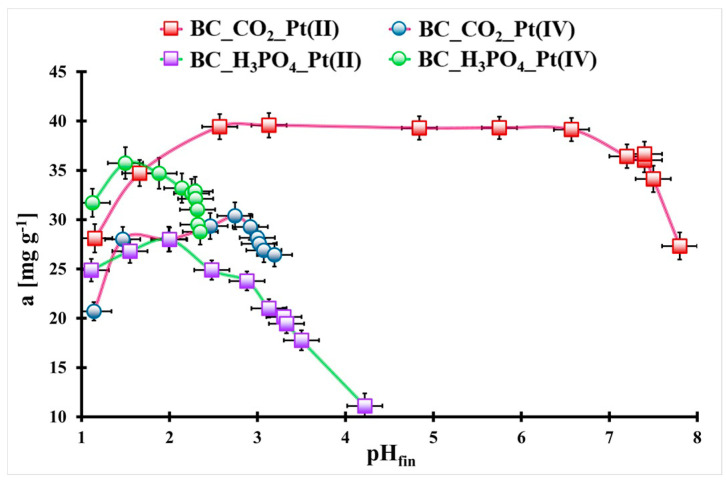
The pH_fin._ influence on Pt(II) and Pt(IV) adsorption onto both studied biochars (Pt(II): m_biochar_ = 35 mg, V_sol._ = 5 mL, t_eq_ = 7 days, C_0_ = 159 mg L^−1^; Pt(IV): m_BC_CO2_ = 20 mg, m_BC_H3PO4_ = 25 mg, V_sol._ = 5 mL, t_eq_ = 7 days, C_0_ = 195 mg L^−1^, T = (20 ± 4) °C). Error bars denote standard deviations from 3 repeats.

In the Figure 3, the pH_fin._ influence on the Pt(II) and Pt(IV) ions adsorption onto the studied biochars is presented. For the BC_CO_2_ biochar, the Pt(II) ions adsorption is the highest and constant in the pH_fin._ range 2.5–6.5. When the pH_fin._ value is out of this range, the Pt(II) adsorption value decreases (from ca. 40 mg g^−1^ to ca. 27 mg g^−1^). Another trend is observed in the case of Pt(IV) ions adsorption onto the same material. In the pH_fin._ range 1.5–2.8, the Pt(IV) adsorption is the highest and stable, but for 1.5 < pH_fin._ > 2.8, the adsorption value of these ions drops from ca. 30 mg g^−1^ to ca. 21 mg g^−1^ (pH_fin._ < 1.5) and to 24 mg g^−1^ (pH_fin._ > 2.8). In both cases, the highest adsorption value of Pt(II) and Pt(IV) onto the BC_CO_2_ biochar surface is observed for the hydroxy- and aqua-complexes of Pt(II) and Pt(IV), which are dominating the Pt(II) and Pt(IV) species in an aqueous solution at pH > 2 or > 1.5, respectively. They possess a slightly negative (e.g., PtCl_3_(H_2_O)^−^, PtCl_5_(OH)^−^) or neutral charge (e.g., PtCl_2_(H_2_O)_2_, PtCl_3_(OH)_2_(H_2_O)), but the surface of the BC_CO_2_ biochar is slightly negatively charged. Probably, the adsorption of Pt(II) and Pt(IV) species could be made possible by the change in the biochar surface charge from negative to positive due to proton adsorption (Figure S1) and electrostatic attraction and/or via the surface complexation [28,29].

In the case of the BC_H_3_PO_4_ biochar, for Pt(II) and Pt(IV) ions, there is a maximum adsorbed value at pH_fin._ = 2.0 or 1.5, respectively. At a pH_fin._ different from 2.0 or 1.5, the Pt(II) or Pt(IV) adsorption onto the BC_H_3_PO_4_ biochar surface drops up to 11 mg g^−1^ (pH_fin._ = 4.2) or 27 mg g^−1^ (pH_fin._ = 2.3), respectively. This suggests that the PtCl_4_^2−^ and PtCl_6_^2−^ complexes are better adsorbed by the BC_H_3_PO_4_ surface than by the BC_CO_2_. The Pt(II) and Pt(IV) species are dominating at pH = 1.5–2.0 [28,29]. The surface of the BC_H_3_PO_4_ is more negatively charged than the BC_CO_2_; thus, its protonation at a low pH could be stronger (Figure S1) and the electrostatic attraction of the negatively charged platinum complexes could be higher. Also, the surface complexation could take place during Pt(II) and Pt(IV) adsorption onto the BC_H_3_PO_4_ surface. For further studies, the Pt(II) and Pt(IV) solutions’ pH was adjusted to 1.5. 

**Figure 4:**
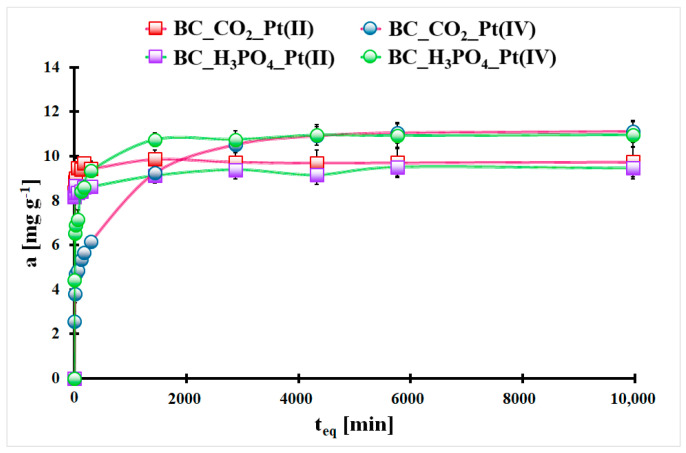
The adsorption kinetics of Pt(II) and Pt(IV) ions onto the studied biochars (m_biochar_ = 20 mg, V_sol._ = 5 mL, C_0_Pt(II)_ = 38.8 mg L^−1^, C_0_Pt(IV)_ = 46.5 mg L^−1^, T = (20 ± 4) °C). Error bars denote standard deviations for 3 repeats.

In Figure 4, the adsorption kinetics of Pt(II) and Pt(IV) ions onto the studied biochars are presented. The Pt(II) adsorption equilibrium onto the BC_CO_2_ and BC_H_3_PO_4_ materials was achieved after 5 min in both cases. In contrast, the Pt(IV) adsorption equilibrium onto the CO_2_-activated biochar and H_3_PO_4_-activated biochar was obtained after 2880 min or 1440 min, respectively. It is obvious that the adsorption of Pt(II) ions onto both studied materials is much faster than in the case of Pt(IV) ions. Additionally, the Pt(IV) ions adsorption onto the studied biochars involves a two-step process, with a first fast step. Both materials possess mesopores in their structure, which allows a simple transfer to the surface active sites of the adsorbent. The slower adsorption kinetics of Pt(IV) ions than Pt(II) ions onto the studied biochars can be the result of the chemisorption reactions between the active surface sites of the studied materials and Pt(IV) ions. They can be complexed by the carbonaceous surface functionalities. 

In Figure S2 and Table 3, there are the results of the experimental kinetics fitting to two theoretical kinetics models (pseudo-second-order (Equation (1)) and Elovich (Equation (2)) [20]). These models are expressed by the following equations:

(1) $$\frac{1}{q_{t}}=\frac{1}{q_{eq}}+\frac{1}{q_{eq}^{2}k_{2}t}$$

(2) $$q_{t}=\frac{1}{b}\ln\left(ab\right)+\frac{1}{b}lnt$$

where *q_t_* is the Pt(II) or Pt(IV) adsorption value at time t [mg g^−1^], *q_eq_* is the Pt(II) or Pt(IV) adsorption capacity at an equilibrium state [mg g^−1^], *k*_2_ is the pseudo-second-order rate constant [g mg^−1^ min^−1^], t is the adsorption time [min], *a* is the initial adsorption rate [mg g^−1^ min^−1^] and *b* is the constant related to the chemisorption activation energy [g mg^−1^]. For Pt(II) adsorption onto the BC_H_3_PO_4_ and Pt(IV) adsorption onto both the studied biochars, the kinetics data are better fitted to the Elovich model (R^2^: 0.976–0.996). In contrast, the Pt(II) adsorption onto the BC_CO_2_ biochar is best fitted to the pseudo-second-order model (R^2^: 0.995). In both models, the chemisorption is the main force influencing the adsorption kinetics. The much lower value for Pt(IV) than Pt(II) is evidence of a slower chemisorption process during Pt(IV) adsorption onto the studied biochars than for Pt(II) ions, but the activation energy (related to the b value) is higher in the case of Pt(II) ions adsorption onto the studied biochars.

**Figure 5:**
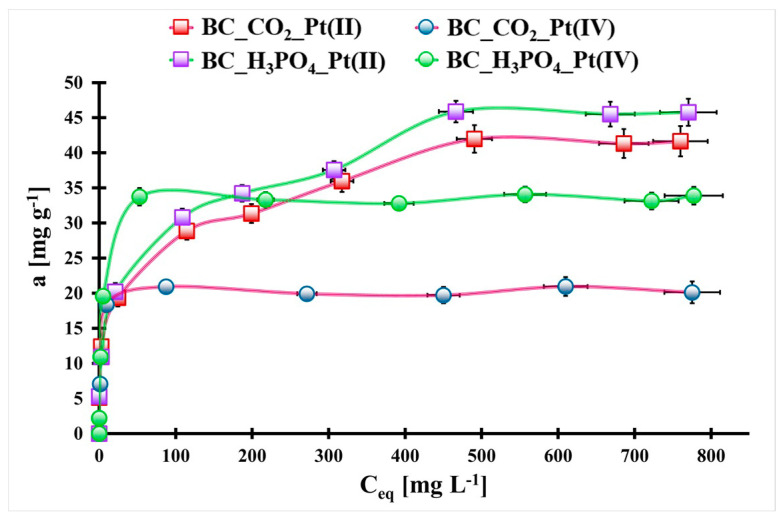
The adsorption isotherms of Pt(II) and Pt(IV) ions onto the studied biochars (m_biochar_ = 20 mg, V_sol._ = 5 mL, pH_0_ = 1.5, t_eq_ = 7 days, T = (25 ± 4) °C). Error bars denote standard deviations for 3 repeats.

In Figure 5, the Pt(II) and Pt(IV) adsorption isotherms of the studied materials are presented. The highest static adsorption capacities for Pt(II) and Pt(IV) ions were estimated for the BC_H_3_PO_4_ biochar: 47 mg g^−1^ and 35 mg g^−1^, respectively. Additionally, higher adsorption values for relatively low equilibrium concentrations were observed for Pt(IV) ions onto both the studied biochars, unlike Pt(II) ions. Thus, both biochars can be used for the efficient Pt(IV) ion removal from aqueous solutions. 

In Figure S3 and Table 4 can be seen the fitting results of the experimental Pt(II) and Pt(IV) adsorption isotherms with two theoretical models: Langmuir (Equation (3)) and Freundlich (Equation (4)) [20]. These models are expressed by the following equations:

(3) $$q_{eq}=\frac{C_{eq}q_{m}k_{L}}{1+C_{eq}k_{L}}$$

(4) $$q_{eq}=k_{F}{C_{eq}}^{n_{F}}$$

where *q_eq_* is the Pt(II) or Pt(IV) adsorption capacity at an equilibrium state [mg g^−1^], *q_m_* is the maximum static Pt(II) or Pt(IV) adsorption capacity [mg g^−1^], *C_eq_* is the Pt(II) or Pt(IV) equilibrium concentration [mg L^−1^], *k_L_* is the Langmuir equilibrium constant [L mg^−1^], *k_F_* is the Freundlich equilibrium constant [mg^1−nF^ L^nF^ g^−1^] and *n_F_* is the Freundlich constant. The Pt(II) adsorption isotherms onto both the studied materials are better described by the Freundlich model (R^2^: 0.859–0.994). This suggests that Pt(II) ions adsorption onto both biochars and Pt(IV) ions onto the BC_CO_2_ material entails the multilayer process onto energetically heterogeneous sites. The *n_F_* values in this case are 0.12–0.25, which confirm the heterogeneity of the surface active sites of the studied materials. On the contrary, the Pt(IV) adsorption isotherms onto the BC_H_3_PO_4_ biochar is best fitted to the Langmuir model (R^2^: 0.996). This means that Pt(IV) adsorption onto this material is mainly controlled by chemisorption and is monolayered adsorption [30]. The BC_H_3_PO_4_ biochar was selected for the further investigations due to its excellent adsorption properties toward Pt(II) and Pt(IV) ions.

### 2.3. Pt Desorption Studies

**Figure 6:**
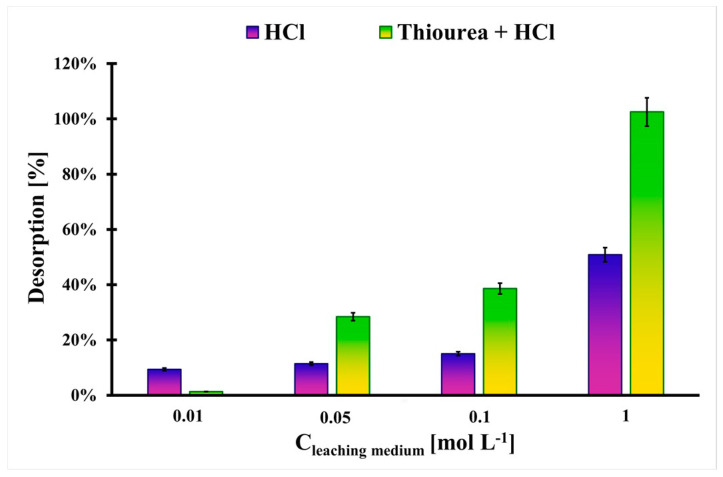
The desorption results of Pt from the Pt-loaded_BC_H_3_PO_4_ with using various liquid media (m_Pt-loaded_BC_H3PO4_ = 10 mg, V_med_ = 4 mL, a_Pt_ = 17.2 mg g^−1^, t_eq_ = 7 days, T = (20 ± 4) °C). Error bars denote standard deviations for 3 repeats.

In Figure 6, the results of Pt desorption from the Pt-loaded_BC_H_3_PO_4_ to the various liquid media are presented. It can be seen that the 1 mol L^−1^ HCl is not efficient for Pt desorption from the studied material (D = 58%). Another situation is observed in the case of the mixture of thiourea and HCl. Their concentration of 1 mol L^−1^ is suitable for quantitative Pt desorption from the BC_H_3_PO_4_ biochar. Thiourea possesses sulfur and ammonia functionalities, which can strongly complex the Pt, and it can overcome impacts that come from the BC_H_3_PO_4_ surface [28]. This medium allows for Pt recovery from the adsorbent and for its regeneration and reuse, which is highly recommended for newly developed adsorbents. 

### 2.4. Adsorption Mechanism

**Figure 7:**
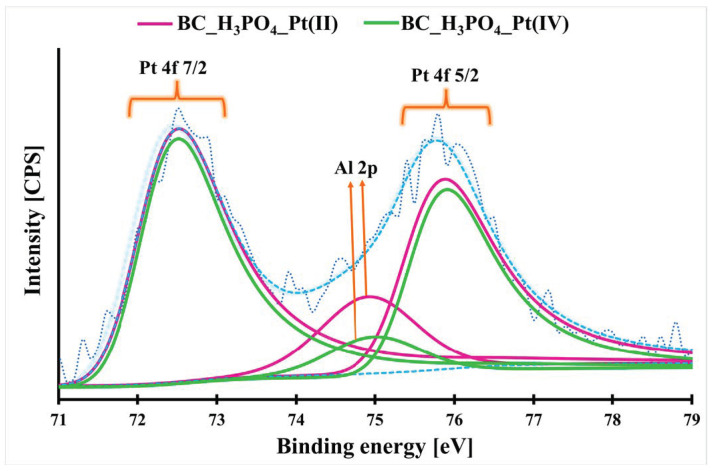
The high-resolution Pt 4f spectra of the Pt-loaded_BC_H_3_PO_4_ biochar. The blue dashed lines represent the background and refined signal, while the dark blue dashed line shows the raw, unsmoothed signal.

More precise investigation of the Pt(II) and Pt(IV) adsorption mechanism onto the BC_H_3_PO_4_ biochar is made possible by studying the high-resolution XPS spectra of the Pt-loaded_BC_H_3_PO_4_ material. For two Pt-loaded_BC_H_3_PO_4_ materials (one after Pt(II) adsorption and another after Pt(IV) adsorption), similar high-resolution Pt 4f spectra were obtained (Figure 7). Both spectra possess one doublet: Pt 4f_7/2_ located at 72.16–72.17 eV and Pt 4f_5/2_ located at 75.52–75.57 eV. In each case, the location of the Pt 4f doublet suggests the formation of PtS on the biochar surface [31,32]. This was evidenced by the high-resolution S 2p spectra. It means that during Pt(II) and Pt(IV) adsorption onto the BC_H_3_PO_4_ biochar, the surface precipitation of PtS can take place. Additionally, the Pt(IV) ions are probably reduced to Pt(II) during the adsorption process. Comparing the C 1s, O 1s and N 1s high-resolution spectra for the pristine BC_H_3_PO_4_ and Pt-loaded_BC_H_3_PO_4_, it can be seen that some changes can take place during Pt(II) and Pt(IV) adsorption process. According to the C 1s spectra, it can be stated that during Pt(II) and Pt(IV) adsorption onto the BC_H_3_PO_4_ biochar, the content of the aromatic carbon rings, carbonyl and carboxyl groups increased and the content of the aliphatic carbon and C-O groups decreased. From the O 1s spectra, it can be stated that the Pt(II) and Pt(IV) adsorption process is related to the decomposition of P-OH and phenol groups and simultaneously the formation of P=O and C-O groups. Additionally, the quaternary nitrogen content decreased and primary/secondary amines content increased during Pt(II) and Pt(IV) adsorption onto the studied biochar. Taking into account these XPS data and the above-mentioned considerations, it can be stated that the adsorption mechanism of Pt(II) and Pt(IV) ions onto the BC_H_3_PO_4_ biochar is complex and can be based on electrostatic attraction, surface complexation, surface precipitation and reduction-oxidation reactions. Further studies are necessary to solve this combined adsorption mechanism. 

### 2.5. Pt(IV) Removal from Catalyst-Originated Extract

**Figure 8:**
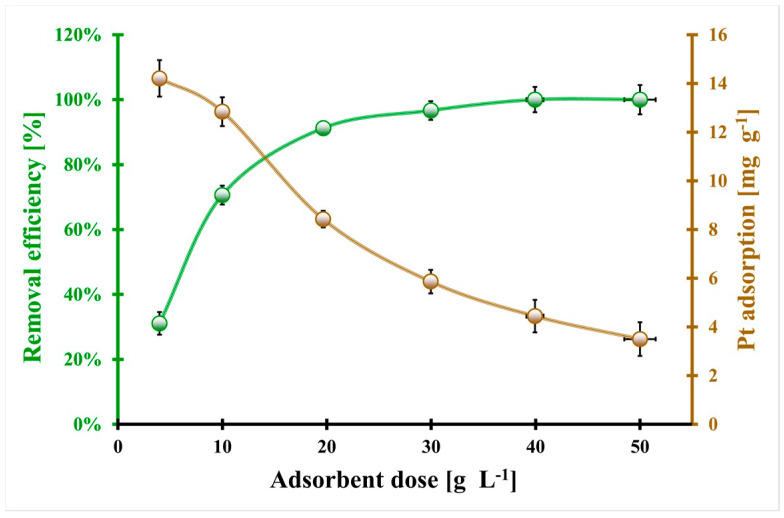
The effect of the BC_H_3_PO_4_ biochar dosage on the platinum removal efficiency and platinum adsorption capacity (pH_fin_ = 1.5, C_0Pt_ = 148.2 mg L^−^^1^, t_eq_ = 3 days, T = (20 ± 4) °C). Error bars denote standard deviations for 3 repeats.

In Figure 8, the platinum removal efficiency and adsorption capacity of platinum in the function of the BC_H_3_PO_4_ biochar dosage are depicted. At higher doses of the adsorbent, the capacity to adsorb platinum decreases significantly, going from 14.20 mg g^−^^1^ to 3.5 mg g^−^^1^. On the contrary, the platinum removal efficiency initially increases from about 31% to 100% within the range of biochar dosage of 4–30 g L^−^^1^, after which it remains constant. The adsorption capacity of platinum decreases as the dosage of biochar increases, which could be attributed to the cohesive interaction between the particles of the biochar, resulting in aggregation or agglomeration [33,34]. Simultaneously, there is a decrease in the effective surface area per unit weight (g) of the adsorbent. In contrast, the initial increase in platinum removal from the leaching solution can be attributed to the introduction of increasing amounts of biochar, which provide a larger surface area for platinum species to interact with. However, beyond a certain point, increasing the biochar dosage has no significant effect on the platinum removal efficiency. The optimal dosage of biochar (13.0 g L^−^^1^) corresponds to the maximum achievable platinum removal efficiency, taking into account the highest platinum adsorption capacity. This relatively low optimal dosage of biochar offers several advantages from the economic, environmental, and engineering perspectives. It implies lower production costs, reduced waste generation, and easier construction of platinum removal systems with lower flow resistance.

## 3. Materials and Methods

### 3.1. Materials and Chemicals

The “Miodek” Apiary Farm in Lublin (Poland) was the manufacturer of the starting material (slumgum (RS)) [35].

The following reagents were used throughout all the work: potassium tetrachloroplatinate(II) (>99%), potassium hexachloroplatinate(IV) (>99%), hydrochloric acid (35%, Suprapur), sodium hydroxide (>90%), thiourea (≥99.0%) orthophosphoric acid (85%, p. a.) and standard solution of platinum (1000 mg L^−1^) obtained from Merck (Darmstadt, Germany), European Reference Material ERM^®^-EB504a from BAM. Carbon dioxide and nitrogen (>99%) were obtained from Air Liquide (Kraków, Poland). Double-distilled Milli-Q water from Millipore (Merck, Darmstadt, Germany) was also used.

### 3.2. Slumgum Carbonization Activation

CO_2_ activation: A total of 15 g of the RS material was heated at 350 °C for 1 h under a CO_2_ atmosphere (110 mL min^−1^) in a quartz tubular furnace. After cooling, the material was crumbled and pyrolyzed in the tubular quartz furnace at 750 °C for 7 h under a CO_2_ atmosphere (110 mL min^−1^). The synthesized biochar was washed several times with 1.5 mol L^−1^ HCl and thoroughly washed with distilled water until the measured filtrate pH was close to 7. Finally, the obtained carbonaceous product was dried overnight at 130 °C. The slumgum-based biochar activated by CO_2_ was labeled as BC_CO_2_.

H_3_PO_4_ activation: A total of 15 g of the RS material was immersed in 75 mL of 60 wt. % H_3_PO_4_ for 3.5 h. Next, the mixture of RS material and acid was dried overnight at 200 °C. Then, the dried mixture was pyrolyzed in the quartz tubular furnace under a nitrogen atmosphere (0.9 L min^−1^) according to the following time–temperature procedure: 25–350 °C (10 °C min^−1^), 350–470 °C (3 °C min^−1^), 470–650 °C (10 °C min^−1^) and 650 °C for 4 h. The obtained solid was washed several times with double-distilled water until the absence of PO_4_^3−^ in the filtrate (test with Fe^3+^ ions). Finally, it was dried overnight at 130 °C. This slumgum-based biochar activated by H_3_PO_4_ was labeled as BC_H_3_PO_4_.

### 3.3. Instrumentation

The nitrogen adsorption/desorption isotherms of the studied biochars were obtained with the ASAP 2420 (Micromeritics Inc., Norcross, GA, USA). Measurements were conducted at −196 °C after the degassing of the studied samples at 120 °C in a vacuum for 12 h. The BET surface area (S_BET_), total pore volume (V_T_) and BJH pore diameter (d_BJH_) were estimated from the desorption branch of the nitrogen adsorption/desorption isotherm in each case.

Morphological analysis of the biochar particles and elemental analysis were carried out using the Carl Zeiss Ultra Plus scanning electron microscope (SEM) (Carl Zeiss, Jena, Germany) equipped with an energy dispersive X-ray (EDX) detector BrukerAXS (Bruker, Karlsruhe, Germany). The SEM microscope was also equipped with secondary electron (SE) and backscattered electron (BSE) detectors. All the experiments were conducted under 20 kV acceleration voltage and 5 nA probe current.

The Raman spectra of all the studied biochars were recorded using an inVia Reflex dispersive Raman microscope (Renishaw, Wotton-under-Edge, UK) equipped with an argon laser (wavelength: 514 nm, source power: 20 mW).

The zeta potential of the studied materials was evaluated using a Zetasizer Nano ZS (Malvern Instruments, Malvern, UK). For each sample, 2 mg of biochar material was immersed in 2 mL of 0.001 mol L^−1^ KCl for 24 h before the measurements.

The elemental analysis and detailed studies concerning the surface functionalities presented on the synthesized biochars were conducted using an X-ray photoelectron spectrometer (XPS) (Prevac, Rogów, Poland) equipped with a monochromatic X-ray source (AlK_α_; 1486.6 eV; Gammadata Scienta, Uppsala, Sweden, source power: 450 W) and calibrated toward the C 1s signal at 285 eV.

The pH measurements were carried out using an N517 pH meter (Mero Tronik, Warsaw, Poland).

The platinum in an aqueous phase was determined with the graphite furnace atomic absorption spectrometer (GF AAS) SpectrAA 880Z (Varian, Belrose, Australia) equipped with the deuterium background correction system and platinum hollow cathode lamp (Varian, Australia). The operating parameters for the Pt determination via the GF AAS technique are presented in Table 5.

### 3.4. Pt(II) and Pt(IV) Adsorption Studies

The Pt(II) and Pt(IV) static adsorption experiments were performed at 25 °C. In each case, 20–35 mg of studied biochar was immersed in 5 mL of an aqueous Pt(II) or Pt(IV) solution. The carbonaceous suspension was shaken at 110 rpm for 7 days, except for the adsorption kinetics studies, where the contact time between the solid and liquid phases was in the range of 5–9960 min. The Pt(II) and Pt(IV) adsorption isotherms were evaluated for the initial Pt(II) and Pt(IV) concentrations between 8.8 mg L^−1^ and 856.2 mg L^−1^. After a defined contact time, the biochar was separated from the aqueous solution by means of centrifugation. The initial and final Pt concentrations were determined in the liquid phase via the GF AAS technique.

The adsorbed amount of Pt(II) or Pt(IV) (a [mg g^−1^]) onto the surface of the studied biochar was calculated as follows:

(5) $$a=\frac{\left(C_{0Pt}–C_{eqPt}\right)V_{sol.}}{m_{biochar}}$$

where *C*_0*Pt*_ is the initial Pt(II) or Pt(IV) concentration in an aqueous solution [mg L^−1^], *C_eqP_*_t_ is the final Pt(II) or Pt(IV) concentration in an aqueous solution [mg L^−1^], *V_sol_*_._ is the volume of the aqueous solution containing Pt(II) or Pt(IV) ions [mL] and *m_biochar_* is the biochar mass [mg].

The desorption studies of Pt from the Pt-loaded BC_H_3_PO_4_ material were conducted in two different media: HCl and thiourea + HCl with concentration range 0.01–1.00 mol L^−1^. In each case, 10 mg of Pt-loaded BC_H_3_PO_4_ was shaken with the 4 mL of a liquid medium for 7 days. After that, the liquid phase was separated from the solid phase via centrifugation and the Pt concentration was measured in a liquid phase using the GF AAS technique. The Pt desorption degree (*D* [%]) was calculated as follows:

(6) $$D=\frac{C_{Pt_{des}}V_{med}}{m_{Pt-loaded\;BC\_H3PO4}\;a_{Pt}}100\%$$

where *C_Ptdes_* is the concentration of the desorbed Pt [mg L^−1^], *V_med_* is the volume of the liquid medium [mL], *m_Pt_loaded_BC_H3PO4_* is the mass of the Pt-loaded_BC_H_3_PO_4_ [mg] and *a_Pt_* is the Pt content in the Pt-loaded_BC_H_3_PO_4_ [mg g^−1^].

### 3.5. Adsorption of Pt(IV) by Biochar from Spent Automotive Catalyst Leaching Solution

Studies of the adsorption capacity of Pt(IV) ions on biochar from the solution after leaching from European Reference Material ERM^®^-EB504a were carried out on the BC_H_3_PO_4_ biochar. This material is a mixture of used cordierite-based car catalysts and was taken as a candidate material. After annealing the material at 700 °C for 12 h, it was ground to a particle size <100 μm using a ball mill with stainless steel balls and a swing mill made of stainless steel. Before weighing for the further extraction step, the material was dried for 8 h at 105 °C. According to the certificate, the content of Platinum Group Elements (PGM) in ERM^®^-EB504a is the following: Pt (1414 ± 9) mg kg^−^^1^, Pd (1596 ± 11) mg kg^−^^1^, Rh (210.0 ± 2.2) mg kg^−^^1^. For the extraction of PGE, the procedure described by Paolo Trucillo et al. [1] was applied, leaching at 20 °C with a 7% H_2_O_2_ and 3.3% HCl solution followed by an adsorption step at room temperature using BC_H_3_PO_4_ biochar. After leaching, the solution was filtered and then a fixed portion of biochar was added to 5 mL of extract. After equilibrium was reached, the platinum concentrations were measured using the GF AAS technique. Experimentally, it was found that during the mild condition of extraction cerium, calcium, strontium and lanthanum up to 0.1% are leached to the solution, and influence on adsorption ability of platinum species. The platinum removal efficiencies (*RE* [%]) (Equation (7)) were calculated as follows:

(7) $$RE=\left(\frac{C_{0Pt}{-C}_{eqPt}}{C_{0Pt}}\right)\times 100\%$$

## 4. Conclusions

The high-temperature CO_2_ and H_3_PO_4_ modification of the raw honeycomb biomass resulted in novel biochars with a micro–mesoporous structure and high specific surface area. The surface of the synthesized materials has an acidic character and there are present some heteroatoms (O, N, P, S), which can act as the active sites for Pt(II) and Pt(IV) adsorption from aqueous solutions. 

Pt(II) adsorption equilibrium was established after 5 min onto both synthesized materials, which can be related to the micropores presence and the fast reactions between Pt(II) ions and surface functionalities. In contrast, the adsorption equilibrium state of Pt(IV) ions onto the studied biochars was reached after 24–48 h. This difference can be the result of the larger Pt(IV) complexes than in the case of Pt(II) and the slower reactions between the biochar surface and Pt(IV) ions. Only the adsorption kinetics of Pt(II) ions onto the CO_2_-modified biochar were well characterized by the pseudo-second-order model. For the rest, the Elovich model was the best fitted one.

The highest static adsorption capacities toward Pt(II) (47 mg g^−1^) and Pt(IV) (35 mg g^−1^) were evaluated for the H_3_PO_4_ modified biochar. Pt(II) adsorption isotherms onto both the biochars and Pt(IV) adsorption isotherm onto the CO_2_-activated material were well fitted to the Freundlich model, which suggests multilayer adsorption onto an energetically heterogeneous surface. In the case of Pt(IV) adsorption isotherm onto the H_3_PO_4_-activated biochar, the Langmuir model the best described the adsorption process (monolayered chemisorption). 

The most effective medium for Pt desorption was 1 mol L^−1^ thiourea in 1 mol L^−1^ HCl, which allowed for the quantitative desorption of this element from the H_3_PO_4_-modified biochar. This medium can be successfully used for the regeneration and reuse of the studied adsorbent, which decreases the costs of its utilization. 

The adsorption mechanism of Pt(II) and Pt(IV) ions onto the H_3_PO_4_-modified biochar is complex and can consist of electrostatic attraction, surface complexation, surface precipitation and redox reactions. Further studies should be carried out to take a more detailed look at this combined adsorption mechanism. 

The H_3_PO_4_-modified biochar synthesized for the first time from the honeycomb biomass can be successfully applied for Pt(IV) removal from the spent automotive catalyst leaching solution.

## Figures and Tables

**Table 1:** Porosity parameters (S_BET_, V_T_, d_BJH_), Raman intensity ratio (I_D_/I_G_) and zeta potential values of the studied biochars.

Biochar Symbol	S_BET_ [m^2^ g^−1^]	V_T_ [cm^3^ g^−1^]	d_BJH_ [nm]	I_D_/I_G_ [a. u.]	ζ [mV]
BC_CO_2_	237 ^#^ ± 11 ^$^	0.13 ^#^ ± 0.01 ^$^	3.7 ^#^ ± 0.1 ^$^	1.25 ^#^ ± 0.03 ^$^	−4.22 ^#^ ± 0.01 ^$^
BC_H_3_PO_4_	608 ^#^ ± 25 ^$^	0.37 ^#^ ± 0.02 ^$^	3.9 ^#^ ± 0.1 ^$^	1.32 ^#^ ± 0.04 ^$^	−28.6 ^#^ ± 0.9 ^$^

^#^—mean value from 3 independent measurements, ^$^—standard deviation from 3 independent measurements.

**Table 2:** Elemental composition of the studied biochars.

**Biochar Symbol**	**XPS**					**XRF**					
	**C** **[wt. %]**	**O** **[wt. %]**	**N** **[wt. %]**	**P** **[wt. %]**	**K** **[wt. %]**	**P** **[wt. %]**	**Si** **[wt. %]**	**S** **[wt. %]**	**Cl** **[wt. %]**	**K** **[wt. %]**	**Fe** **[wt. %]**
BC_CO_2_	70.0 ^#^ ± 2.1 ^$^	23.8 ^#^ ± 1.2 ^$^	2.5 ^#^ ± 0.2 ^$^	1.4 ^#^ ± 0.1 ^$^	2.3 ^#^ ± 0.1 ^$^	0.85 ^#^ ± 0.01 ^$^	2.87 ^#^ ± 0.07 ^$^	0.73 ^#^ ± 0.01 ^$^	5.58 ^#^ ± 0.12 ^$^	1.61 ^#^ ± 0.03 ^$^	6.91 ^#^ ± 0.27 ^$^
BC_H_3_PO_4_	57.3 ^#^ ± 2.4 ^$^	27.2 ^#^ ± 3.3 ^$^	4.1 ^#^ ± 0.5 ^$^	11.4 ^#^ ± 1.8 ^$^	nr	13.6 ^#^ ± 1.8 ^$^	1.85 ^#^ ± 0.02 ^$^	nr	nr	nr	1.25 ^#^ ± 0.07 ^$^
**Biochar Symbol**	**SEM-EDS**										
	**C** **[wt. %]**	**O** **[wt. %]**	**N** **[wt. %]**		**P** **[wt. %]**	**K** **[wt. %]**		**Cl** **[wt. %]**	**Mg** **[wt. %]**	**Ca** **[wt. %]**	
BC_CO_2_	83.0 ^#^ ± 3.4 ^$^	9.06 ^#^ ± 1.48 ^$^	3.97 ^#^ ± 0.41 ^$^		0.29 ^#^ ± 0.45 ^$^	0.43 ^#^ ± 0.61 ^$^		1.04 ^#^ ± 0.38 ^$^	0.18 ^#^ ± 0.25 ^$^	0.22 ^#^ ± 0.27 ^$^	
BC_H_3_PO_4_	55.6 ^#^ ± 3.2 ^$^	26.7 ^#^ ± 3.3 ^$^	4.25 ^#^ ± 0.27 ^$^		10.3 ^#^ ± 0.6 ^$^	0.22 ^#^ ± 0.09 ^$^		nr	0.12 ^#^ ± 0.05 ^$^	0.25 ^#^ ± 0.13 ^$^	

^#^—mean value from 3 independent measurements, ^$^—standard deviation from 3 independent measurements, nr—not registered.

**Table 3:** Results of the experimental kinetics fitting to the theoretical models (pseudo-second-order (PSO) and Elovich).

**Material** **Symbol**	**Pt(II)**						
	**PSO**				**Elovich**		
	**q_eq,t._** **[mg g^−1^]**	**q_eq,exp._** **[mg g^−1^]**	**k_2_** **[g mg^−1^ min^−1^]**	**R^2^**	**a** **[mg g^−1^ min^−1^]**	**b** **[g mg^−1^]**	**R^2^**
BC_CO_2_	9.63 ^#^ ± 0.06 ^$^	9.86 ^#^ ± 0.37 ^$^	0.12 ^#^ ± 0.02 ^$^	0.995	3.3 × 10^23 #,^*	6.56 ^#^ ± 0.26 ^$^	0.993
BC_H_3_PO_4_	8.96 ^#^ ± 0.13 ^$^	9.51 ^#^ ± 0.21 ^$^	0.17 ^#^ ± 0.08 ^$^	0.976	1.2 × 10^18 #,^*	5.57 ^#^ ± 0.13 ^$^	0.996
**Material** **Symbol**	**Pt(IV)**						
	**PSO**				**Elovich**		
	**q_eq,t._** **[mg g^−1^]**	**q_eq,exp._** **[mg g^−1^]**	**k_2_** **[g mg^−1^ min^−1^]**	**R^2^**	**a** **[mg g^−1^ min^−1^]**	**b** **[g mg^−1^]**	**R^2^**
BC_CO_2_	10.4 ^#^ ± 0.7 ^$^	11.1 ^#^ ± 0.5 ^$^	0.001 *	0.850	1.30 ^#^ ± 0.01 ^$^	0.81 ^#^ ± 0.04 ^$^	0.976
BC_H_3_PO_4_	10.3 ^#^ ± 0.3 ^$^	10.9 ^#^ ± 0.3 ^$^	0.009 *	0.921	92.9 ^#^ ± 6.4 ^$^	1.19 ^#^ ± 0.08 ^$^	0.976

^#^—mean value from 3 independent measurements, ^$^—standard deviation from 3 independent measurements, *—SD close to 0.

**Table 4:** Results of the experimental adsorption isotherm fitting to the theoretical models (Langmuir and Freundlich).

**Material** **Symbol**	**Pt(II)**						
	**Langmuir**				**Freundlich**		
	**q_max,t._** **[mg g^−1^]**	**q_max,exp._** **[mg g^−1^]**	**k_L_** **[L mg^−1^]**	**R^2^**	**n_F_** **[a. u.]**	**k_F_** **[mg^1−nF^ L^nF^ g^−1^]**	**R^2^**
BC_CO_2_	41.1 ^#^ ± 2.8 ^$^	42.0 ^#^ ± 1.8 ^$^	0.03 ^#^ ± 0.01 ^$^	0.917	0.22 ^#^ ± 0.01 ^$^	9.99 ^#^ ± 0.68 ^$^	0.994
BC_H_3_PO_4_	45.0 ^#^ ± 2.7 ^$^	45.9 ^#^ ± 2.2 ^$^	0.03 ^#^ ± 0.01 ^$^	0.939	0.25 ^#^ ± 0.01 ^$^	9.36 ^#^ ± 0.83 ^$^	0.991
**Material** **symbol**	**Pt(IV)**						
	**Langmuir**				**Freundlich**		
	**q_max,t._** **[mg g^−1^]**	**q_max,exp._** **[mg g^−1^]**	**k_L_** **[L mg^−1^]**	**R^2^**	**n_F_** **[a. u.]**	**k_F_** **[mg^1−nF^ L^nF^ g^−1^]**	**R^2^**
BC_CO_2_	24.2 ^#^ ± 2.9 ^$^	20.9 ^#^ ± 0.3 ^$^	0.31 ^#^ ± 0.07 ^$^	0.666	0.12 ^#^ ± 0.03 ^$^	9.81 ^#^ ± 1.89 ^$^	0.859
BC_H_3_PO_4_	34.0 ^#^ ± 0.4 ^$^	34.1 ^#^ ± 1.5 ^$^	0.28 ^#^ ± 0.02 ^$^	0.996	0.15 ^#^ ± 0.03 ^$^	13.2 ^#^ ± 2.7 ^$^	0.878

^#^—mean value from 3 independent measurements, ^$^—standard deviation from 3 independent measurements.

**Table 5:** GF AAS operating parameters during the Pt determination.

Parameter	Value
Lamp current [mA]	10.0
Wavelength [nm]	265.9
Slit width [nm]	0.5
Sample volume [µL]	15
Atomization temperature [°C]	2700
Pyrolysis temperature [°C]	1000

## Data Availability

The raw/processed data required to reproduce these findings cannot be shared at this time due to technical or time limitations.

## Supplementary Materials

The following supporting information can be downloaded at https://www.mdpi.com/article/10.3390/molecules29020547/s1, Figure S1: The pH_fin_ in the function of the pH_in_ for studied adsorption systems (Pt(II): m = 35 mg, V = 5 mL, t = 7 days, C_0_ = 159 mg L^−1^; Pt(IV): m_BC_CO2_ = 20 mg, m_BC_H3PO4_ = 25 mg, V = 5 mL, t = 7 days, C_0_ = 195 mg L^−1^); Figure S2: The experimental Pt(II) and Pt(IV) adsorption kinetics data fitted to the theoretical pseudo-second-order and Elovich models for a) BC_CO_2__Pt(II), b) BC_CO_2__Pt(IV), c) BC_H_3_PO_4__Pt(II) and d) BC_H_3_PO_4__Pt(IV) (m_biochar_ = 20 mg, V_sol._ = 5 mL, C_0_Pt(II)_ = 38.8 mg L^−1^, C_0_Pt(IV)_ = 46.5 mg L^−1^, T = (20 ± 4)°C), error bars denote standard deviations for 3 repeats); Figure S3: The experimental Pt(II) and Pt(IV) adsorption isotherms data fitted to the theoretical Langmuir and Freundlich models for a) BC_CO_2__Pt(II), b) BC_CO_2__Pt(IV), c) BC_H_3_PO_4__Pt(II) and d) BC_H_3_PO_4__Pt(IV) (m_biochar_ = 20 mg, V_sol._ = 5 mL, pH_0_ = 1.5, t_eq_ = 7 days, T = (25 ± 4) °C), error bars denote standard deviations for 3 repeats.
