# Supplementary material for: Optimization of Pt(II) and Pt(IV) Adsorption from a Water Solution on Biochar Originating from Honeycomb Biomass
Source: Molecules. 2024 Jan 22;29(2):547. doi: 10.3390/molecules29020547 (PMC10820625; doi:10.3390/molecules29020547)
Supplement: Supplementary file 1 [file molecules-29-00547-s001.zip › molecules-2812161-supplementary.pdf]

Optimization of Pt(II) and Pt(IV) Adsorption from a Water Solution on Biochar Originating from Honeycomb Biomass

Kinga Morlo [1], Rafał Olchowski [2] and Ryszard Dobrowolski [1,*]

[1] Department of Analytical Chemistry, Institute of Chemical Sciences, Faculty of Chemistry,
Maria Curie-Sklodowska University, M. C. Sklodowska Sq. 3, 20-031 Lublin, Poland; kinga.morlo1@gmail.com

[2] Department of Pharmacology, Toxicology and Environmental Protection, Faculty of Veterinary Medicine, University of Life Sciences, Akademicka Sq. 12, 20-950 Lublin, Poland; rafal.olchowski@up.lublin.pl

* Correspondence: rdobrow@poczta.umcs.lublin.pl

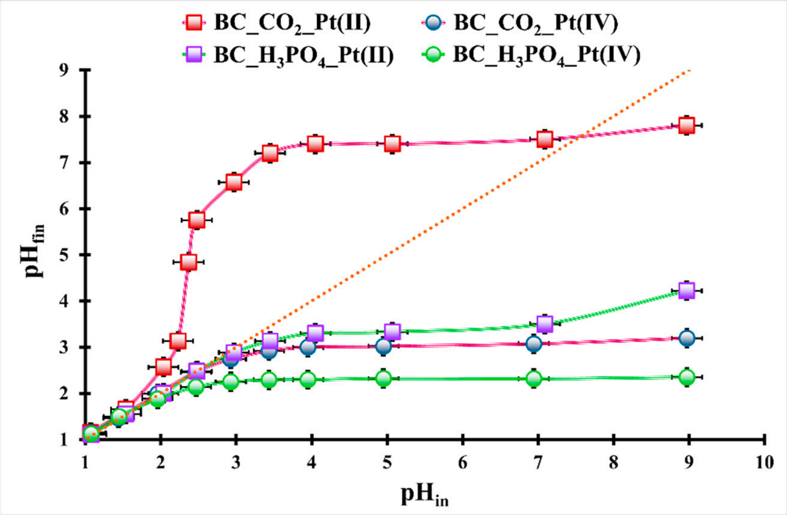

Figure S1. The pH_{fin} in the function of the pH_{in} for studied adsorption systems (Pt(II): m = 35 mg, V = 5 mL, t = 7 days, C_0 = 159 mg L^{-1}; Pt(IV): m_{BC_CO2} = 20 mg, m_{BC_H3PO4} = 25 mg, V = 5 mL, t = 7 days, C_0 = 195 mg L^{-1})

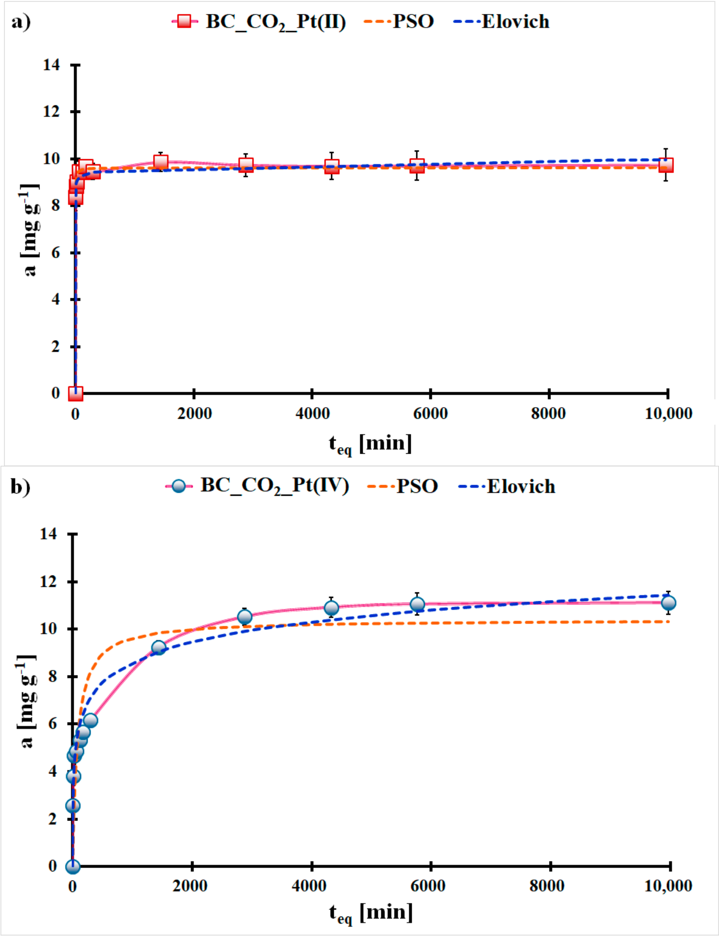

a)
BC_CO2_Pt(II)
PSO
Elovich
a [mg g-1]
14
12
10
8
6
4
2
0
0
2000
4000
6000
8000
10,000
teq [min]
b)
BC_CO2_Pt(IV)
PSO
Elovich
a [mg g-1]
14
12
10
8
6
4
2
0
0
2000
4000
6000
8000
10,000
teq [min]

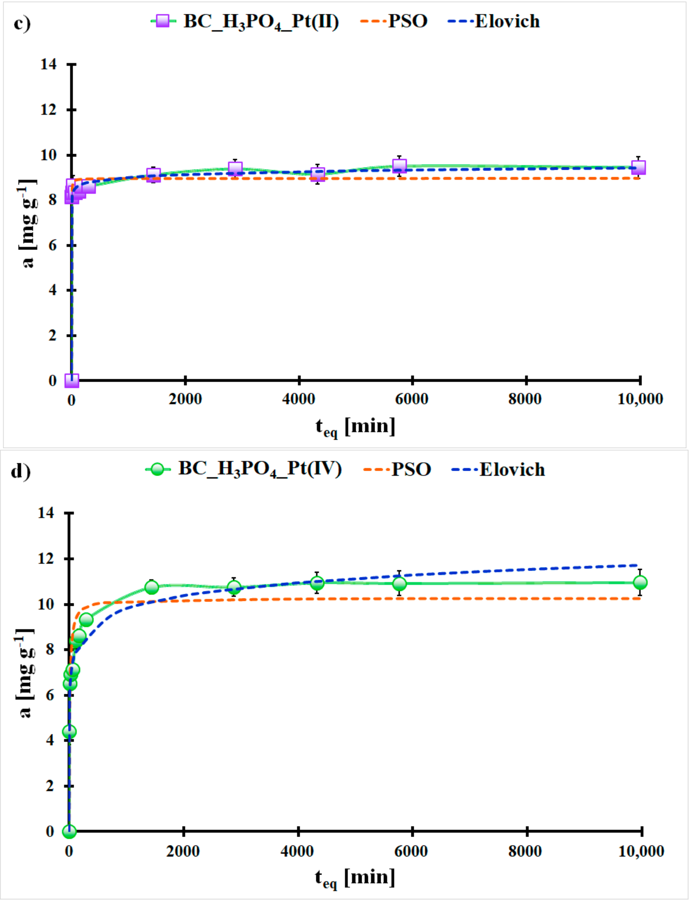

Figure S2. The experimental Pt(II) and Pt(IV) adsorption kinetics data fitted to the theoretical pseudo-second-order and Elovich models for a) $BC\_CO_2\_Pt(II)$, b) $BC\_CO_2\_Pt(IV)$, c) $BC\_H_3PO_4\_Pt(II)$ and d) $BC\_H_3PO_4\_Pt(IV)$ ($m_{biochar}$ = 20 mg, $V_{sol.}$ = 5 mL, $C_{0\_Pt(II)}$ = 38.8 mg L^{-1}, $C_{0\_Pt(IV)}$ = 46.5 mg L^{-1}, T = (20 ± 4)°C), error bars denote standard deviations for 3 repeats)

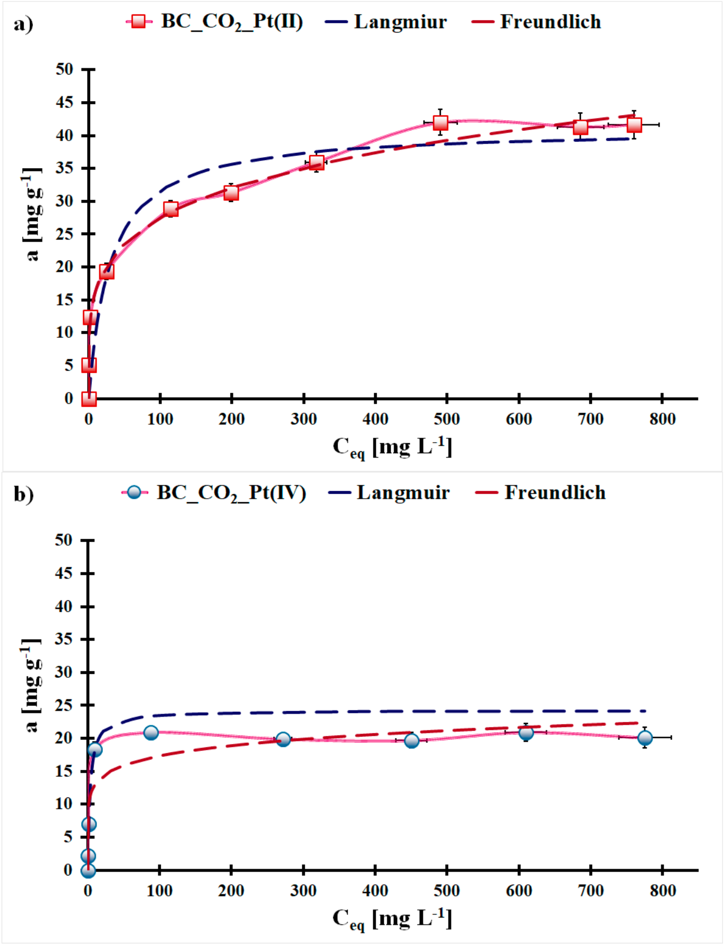

a)
BC_CO2_Pt(II)
Langmiur
Freundlich
a [mg g-1]
0
5
10
15
20
25
30
35
40
45
50
0
100
200
300
400
500
600
700
800
Ceq [mg L-1]
b)
BC_CO2_Pt(IV)
Langmuir
Freundlich
a [mg g-1]
0
5
10
15
20
25
30
35
40
45
50
0
100
200
300
400
500
600
700
800
Ceq [mg L-1]

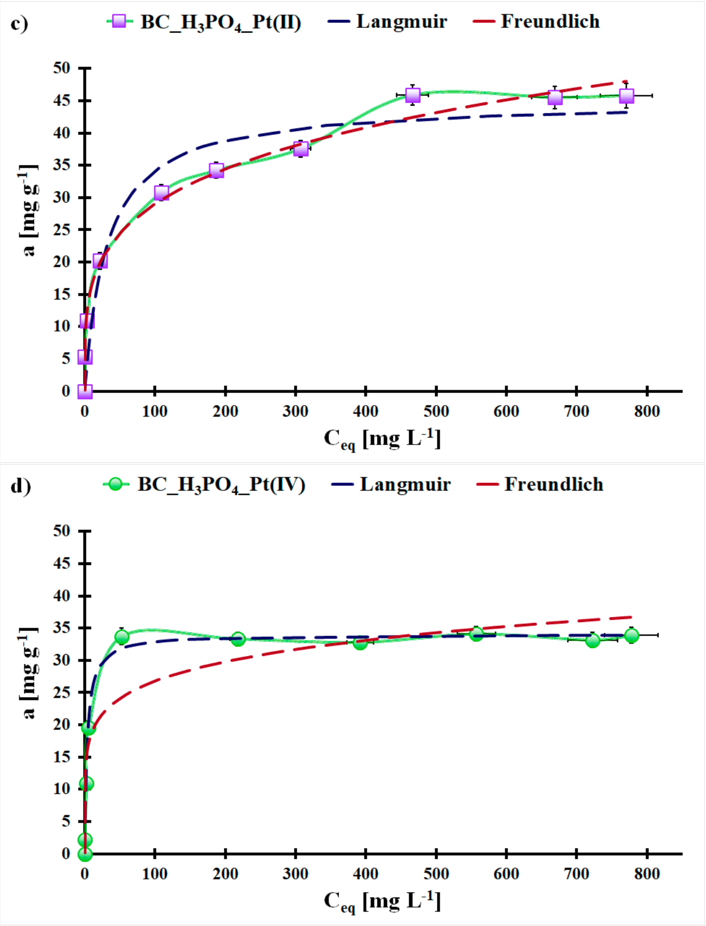

Figure S3. The experimental Pt(II) and Pt(IV) adsorption isotherms data fitted to the theoretical Langmuir and Freundlich models for a) BC_CO_2_Pt(II), b) BC_CO_2_Pt(IV), c) BC_H_3PO_4_Pt(II) and d) BC_H_3PO_4_Pt(IV) ($m_{biochar}$ = 20 mg, $V_{sol.}$ = 5 mL, pH_0 = 1.5, t_{eq} = 7 days, T = (25 ± 4) °C), error bars denote standard deviations for 3 repeats
